# Supplementary material for: Non-Invasive Real-Time Monitoring of Bacterial Activity by Non-Contact Impedance Spectroscopy for Off-the-Shelf Labware
Source: Sensors (Basel). 2025 Apr 11;25(8):2427. doi: 10.3390/s25082427 (PMC12031269; doi:10.3390/s25082427)
Supplement: Supplementary file 1 [file sensors-25-02427-s001.zip › sensors-3508650-supplementary.pdf]

Supplementary Information for

Non-invasive Real-Time Monitoring of Bacterial Activity by Non-contact Impedance Spectroscopy for off-the-shelf labware

Carsten Thirstrup[1], Ole Stender Nielsen[1], Mikael Lassen[1], Thomas Emil Andersen[2,3] and Hüsnü Aslan[1]*

[1] DFM A/S, Kogle Allé 5, DK-2970 Hørsholm, Denmark; cth@dfm.dk; ocn@dfm.dk; ml@dfm.dk; asl@dfm.dk

[2] Department of Clinical Microbiology, Odense University Hospital, Odense, Denmark

[3] Research Unit of Clinical Microbiology, University of Southern Denmark, Odense, Denmark; thandersen@health.sdu.dk

*) Correspondence: asl@dfm.dk, tel. +45 2545 9057

Raman Datasets for machine learning

The Raman dataset consists of 17 classes of bacteria and 1 non-bacteria class:

1. E. coli ATCC 35218 on CaF2
2. E. coli ATCC 25922 on CaF2
3. E. coli Asyp (clinical isolate from urine) on CaF2
4. E. coli Asyp in BHI-medium
5. methicillin-resistant S. epidermidis ATCC 35984 (MRSE) on CaF2
6. methicillin-sensitive S. epidermidis ATCC 14990 (MSSE) on CaF2
7. methicillin-sensitive S. epidermidis ATCC 14990 (MSSE) in BHI-medium
8. Micrococcus luteus on CaF2
9. S. lugdunensis on CaF2
10. S. haemolyticus on CaF2
11. S. pettenkoferi on CaF2
12. S. saprophyticus on CaF2
13. P. aeruginosa on CaF2
14. k. pneumonia on CaF2
15. methicillin-resistant S. aureus ATCC 252 (MRSA) on CaF2
16. methicillin-sensitive S. aureus ATCC 2752 (MSSA) on CaF2
17. C. afermentans (unknown) on CaF2
18. Calcium fluoride slide CaF2

The data of the bacterial classes in the Bacteria-surface training dataset were acquired by measuring over CaF_2 slides, which were completely covered by multilayer bacterial mats. Test samples were prepared separately from samples used for training. To prepare samples for Raman training measurement, a sample was simply transferred from a single colony directly to a sterilized CaF2 Raman-grade objective slide.

Data availability

The data that support the findings of this study are available from the corresponding author upon reasonable request.

Table S1 Comparison of measurement methods.

Aspect	Non-Contact Impedance Spectroscopy (NCIS)	Optical Density (OD) Measurements	Traditional Contact-Based Impedance Spectroscopy (IS)
**Performance**	Provides real-time monitoring of both planktonic and biofilm growth without direct contact with the culture medium	Measures turbidity to estimate planktonic cell concentrations; does not detect biofilms	Monitors changes in electrical properties due to microbial activity; electrodes are in direct contact with the medium
**Sensitivity**	Capable of detecting early-stage biofilm formation[1] and planktonic growth	Effective for moderate to high planktonic cell densities; limited sensitivity to low concentrations	Sensitive to both planktonic and biofilms; however, electrode fouling can affect sensitivity
**Detection limits**	Can detect bacterial concentrations as low as 10^1 CFU/mL, depending on system configuration[2-7]	Typically detects cell concentrations above 10^6 CFU/mL[8]	Can detect bacterial concentrations as low as 10^1 CFU/mL, depending on system configuration[2-6]
**Advantages**	Non-invasive; reduces contamination risk; suitable for continuous monitoring; detects both planktonic and biofilm growth	Simple and cost-effective; widely used; provides rapid results for planktonic cells	Suitable for continuous monitoring; detects both planktonic and biofilm growth
**Disadvantages**	Requires specialized electronics; calibration can be complex	Limited to planktonic cells; cannot detect biofilms; requires sampling, which can introduce contamination; cannot provide real-time monitoring	Requires specialized electronics; calibration can be complex; electrode fouling affects accuracy; direct contact poses contamination risks and regular maintenance

References

1. C. E. Turick, H. Colon-Mercado, C. E. Bagwell, S. D. Greenway and J. W. Amoroso, *SN Applied Sciences*, 2020, **2**, 389.
2. X. Muñoz-Berbel, N. Vigués, M. Cortina-Puig, R. Escudé, C. García-Aljaro, J. Mas and F. X. Muñoz, *Analytical Methods*, 2010, **2**, 1036-1042.
3. X. Muñoz-Berbel, N. Vigués, A. T. A. Jenkins, J. Mas and F. J. Muñoz, *Biosensors and Bioelectronics*, 2008, **23**, 1540-1546.
4. J. Park, Y. Lee, Y. Hwang and S. Cho, *Sensors*, 2020, **20**, 5237.
5. J. Saulnier, C. Jose and F. Lagarde, *Bioelectrochemistry*, 2024, **155**, 108587.
6. A. K. Shukla, J. S. Boruah, S. Park and B. Kim, *The Journal of Physical Chemistry C*, 2024, **128**, 13458-13463.
7. W. Stilman, M. Campolim Lenzi, G. Wackers, O. Deschaume, D. Yongabi, G. Mathijssen, C. Bartic, J. Gruber, M. Wübbenhorst, M. Heyndrickx and P. Wagner, *physica status solidi (a)*, 2022, **219**, 2100405.
8. R. J. H. Hammond, K. Falconer, T. Powell, R. Bowness and S. H. Gillespie, *Scientific Reports*, 2022, **12**, 19393.
